# Commercializing Personal Health Information: A Critical Qualitative Content Analysis of Documents Describing Proprietary Primary Care Databases in Canada

**DOI:** 10.34172/ijhpm.2023.6938

**Published:** 2023-05-02

**Authors:** Sheryl Spithoff, Quinn Grundy

**Affiliations:** ^1^Department of Family and Community Medicine, University of Toronto, Toronto, ON, Canada; ^2^Department of Family and Community Medicine, Women’s College Hospital, Toronto, ON, Canada; ^3^Women’s College Research Institute, Women’s College Hospital, Toronto, ON, Canada; ^4^Lawrence S. Bloomberg Faculty of Nursing, University of Toronto, Toronto, ON, Canada

**Keywords:** Health Data, Commercialization, Privacy, Pharmaceutical Industry, Primary Care, Canada

## Abstract

**Background:** Commercial data brokers have amassed large collections of primary care patient data in proprietary databases. Our study objective was to critically analyze how entities involved in the collection and use of these records construct the value of these proprietary databases. We also discuss the implications of the collection and use of these databases.

**Methods:** We conducted a critical qualitative content analysis using publicly available documents describing the creation and use of proprietary databases containing Canadian primary care patient data. We identified relevant commercial data brokers, as well as entities involved in collecting data or in using data from these databases. We sampled documents associated with these entities that described any aspect of the collection, processing, and use of the proprietary databases. We extracted data from each document using a structured data tool. We conducted an interpretive thematic content analysis by inductively coding documents and the extracted data.

**Results:** We analyzed 25 documents produced between 2013 and 2021. These documents were largely directed at the pharmaceutical industry, as well as shareholders, academics, and governments. The documents constructed the value of the proprietary databases by describing extensive, intimate, detailed patient-level data holdings. They provided examples of how the databases could be used by pharmaceutical companies for regulatory approval, marketing and understanding physician behaviour. The documents constructed the value of these data more broadly by claiming to improve health for patients, while also addressing risks to privacy. Some documents referred to the trade-offs between patient privacy and data utility, which suggests these considerations may be in tension.

**Conclusion:** Documents in our analysis positioned the proprietary databases as socially legitimate and valuable, particularly to pharmaceutical companies. The databases, however, may pose risks to patient privacy and contribute to problematic drug promotion. Solutions include expanding public data repositories with appropriate governance and external regulatory oversight.

## Background

**Key Messages**

** Implications for policy makers**

- Commercial entities have databases comprised of millions of de-identified Canadian primary care records.
- These databases help pharmaceutical companies to demonstrate the safety and efficacy of their products in real world situations, market their products, and understand physician behaviour.
- Regulator and funder interest in using “real world data” — data collected outside of clinical trials — to demonstrate safety and efficacy of new pharmaceuticals may be driving the use of these proprietary databases.
- These databases, however, may present risks to privacy; enable surveillance and microtargeting of patients who share similar characteristics; and contribute to problematic drug promotion.
- Solutions could include expanding public data repositories with diverse governance and external regulatory oversight.

** Implications for the public**

Commercial data brokers collect de-identified primary care patient health records from around the world, including 1.2 million records from Canada. In our study we analyzed documents describing the collection and uses of these data in the Canadian context. We found that documents contained tensions such as claiming that data uses benefit society, while also showing how pharmaceutical companies use the data to market their products, an activity known to cause harm. The documents also claim that privacy is never compromised, while also describing how the databases contain patient-level records with large amounts of sensitive medical details. These risks highlight the issue of consent to use the patient data, which is currently granted by the physicians who collect the data during the provision of care. However, if patients are at risk of harms, physician consent may not be adequate. We recommend implementing processes to enable societal benefits from patient data, while addressing risks and ethical concerns.

Over the past few decades, commercial data brokers (ie, for-profit companies that aggregate, analyze and monetize personal information) have amassed large collections of patient data.^1-3^ IQVIA, a health data giant, claims to have 530 million de-identified patient records from 24 different countries, including 1.2 million primary care records from Canada.^2-6^ Primary care patient records are a highly sought-after type of patient data^7^ with rich, contextual and longitudinal information.^8,9^ Pharmaceutical companies are the health data broker industry’s main customer.^3^ They use the data for market research, drug development, marketing, and monitoring drug adherence.^2,10,11^ Other customers include the insurance and artificial intelligence industries, governments, academics, and non-profit research organizations. These entities use the data for a variety of reasons, from creating new artificial intelligence technologies to research and public health initiatives.^5,12-15^ This collection of data is not unique to the health industry, but is part of an economic system that increasingly depends on the mass collection and analysis of data.^16-19^ The “Big Tech” companies that embrace this model (eg, Meta, Alphabet Inc.) dominate world markets and contemporary capitalism.^17,18,20,21^

 In addition to commercial and research opportunities, the secondary uses of patient data also present risks.^3,22,23^ One risk is loss of anonymity from re-identification.^24,25^ If data were truly anonymized, with no re-identification risk, they would have little value because most useful information (eg, age, general location, gender etc) would be removed.^7,26-28^ As a result, re-identification of some individuals is always possible.^29^ The risk of re-identification is more likely for individuals who have rare conditions or whose health problems have been reported in the media (eg, public figures, victims of motor vehicle collisions).^30^ Another risk is the use of de-identified and aggregated data for commercial gain that may be at odds with community or population health and well-being.^3,31^

 Despite the risks presented by the collection and secondary uses of de-identified patient data, these data receive few protections. Under current federal and provincial privacy legislation in Canada, de-identified data fall outside the scope of the law.^32,33^ Further, a recent ruling by the Ontario Privacy Commissioner, states that companies do not need to seek patient consent to de-identify their personal health information (a subset of personal information pertaining to an individual’s health). They are required, however, to provide a public notice describing how the data will be used.^23,24^

 To date, the health data broker industry has received little attention in the media and research literature.^1,35-39^ Documents from entities involved in the collection, creation and use of proprietary databases, therefore, provide an opportunity to explore social practices that are not widely known nor readily observable.^40^ The documents can provide insight into how these data are valued by data users, as well as the ethical issues and the risks to patients, communities, and society. The messages in the documents may, in turn, affect how these risks and benefits are understood, shaping discourse and influencing policy.^40^ Thus, we sought to sample documents produced by entities involved in the collection and use of proprietary databases of primary care patient records in the Canadian context.

 Although the collection and use of de-identified patient data without patient consent is legal in Canada, as in most countries,^41^ these practices may not be aligned with the views of the public, who are generally opposed to commercial entities controlling their data.^42-45^ These types of documents, therefore, can function as statements of legitimacy, used to demonstrate that an action is beneficial, ethical and socially acceptable.^44,46-49^ They are meant to reassure customers, shareholders and regulators, as well as to influence public discourse and policy-makers.^50-52^ These claims to legitimacy may affect how benefits and risks are understood and in turn affect political and academic discourses.

 Our research objective, therefore, was to understand the main messages in documents and analyze how they construct the value of proprietary primary care patient databases. We sampled proprietary databases containing primary care records from Canada having identified publicly available documents describing these databases and their uses.^35^ We sought to understand the texts within their social context, and, in our discussion, we provide an understanding of who these claims might benefit and the broader societal implications.

## Methods

 We conducted a critical content analysis of publicly available documents produced by entities involved in the collection, processing, storage and end-use of the proprietary primary care patient databases. Critical qualitative content analysis is a methodology that uses documents as the primary data source.^40,53-55^ Documents are an underused source of information, often relegated to supporting roles in qualitative studies, but contain rich content and contextual information allowing them to function as a primary data source. Qualitative content analyses, as a methodology, addresses content, context, credibility and audience. The analysis relies on theoretical presuppositions and purposive sampling with deeper, repetitive readings of the texts to identify patterns, meanings and themes in the documents.^40,53-55^ Critical approaches understand texts as value-laden and situated within a specific social context and power structures.^40,54,56^ Additionally, critical approaches are action-oriented, arguing that knowledge generation should address social order, in particular, “oppressive social structures.”^57^ Critical content analyses have been used to analyze police training materials,^58^ media reporting on the opioid crisis,^59^ and corporate promotional materials.^55,60^ The study authors have expertise in qualitative methods, critical content analysis, discourse analysis, health policy, digital technologies, and the interactions between commercial entities and the healthcare system. We reported our methods using the Standards for Reporting Qualitative Research^61^ (Supplementary file 1).

###  Data Sources and Sampled Entities

 Using structured internet searches (Supplementary file 2), we identified commercial health data brokers (ie, for-profit companies that aggregate, analyze and monetize personal information) operating in Canada in the past 10 years. Search terms included “de-identify,” “Canada,” “primary care,” “electronic medical records,” and “real world evidence.” For each commercial data broker, we identified their subsidiaries and proprietary databases containing primary care data from people living in Canada. We then ran structured, systematic internet and electronic database searches to identify entities involved in the collection, processing, aggregation and end-use of these proprietary databases.

###  Inclusion and Exclusion Criteria

 We used a criterion typology for purposive sampling,^62^ where we included all documents that described any aspect of the collection, processing and end-use of the proprietary databases with Canadian primary care data according to a pre-specified set of criteria. For entities identified through structured Google searches, we identified relevant, publicly available documents through further internet and systematic database searches conducted between March 2021 and August 2021. When searches returned results, we included the web page or document (eg, reports, posters, slide decks), if they were associated with the sampled entities.

 We considered a document to be associated with a sampled/selected entity if it was:

1. a webpage on the sampled entity’s official website;
2. a document located on the sampled entity’s official website and branded with a logo or copyright statement;
3. a document authored by a current employee of one of the sampled entities.

We did not sample documents describing data sourced from outside Canada; data sourced from acute care settings; or in languages other than English.

###  Data Collection

 We created a structured, open-ended data extraction form based on past research, which aimed to understand how health data are transformed into proprietary databases.^1,31,37,63^ The form included document source information, such as author, date of creation, type of document (eg, presentation, abstract, document on company website) and intended audience. We determined intended audience by coding for statements in the document that provided insight into the target audience. We coded for explicit statements addressing an audience, and implicit statement indicating an intended audience (eg, describing how a particular group could benefit from the data). The extraction form contained sections on patient data sources/collection, consent, de-identification, sale/purchase of data, data storage, data innovation, data validation, end-uses of data, ownership, and control of data. SS tested the form on several documents and together with QG revised the document. SS then extracted data from each document.

###  Data Analysis 

 We closely read each source document and the accompanied extracted data. We created memos offering interpretation, examining socially situated meanings, and identifying lines of inquiry. Consistent with a critical approach, the focus of our analysis was to provide an understanding of who these claims might benefit, how they uphold current power structures and what harms/risks are ignored. SS maintained an audit trail to record the research path, including observations, discussions, decisions, and activities. We uploaded all source documents into NVivo. Based on these interpretive memos, SS constructed a preliminary coding tree and reviewed with QG to identify important concept areas and emerging themes. SS continued to use memos to record thought processes, decisions, and uncertainties throughout coding. Using the refined codebook, SS coded the rest of the documents, while meeting frequently with QG to review findings. As the analysis progressed, SS wrote interpretive memos based on the codes and SS and QG reviewed these memos collectively to develop preliminary concept areas and themes. After analyzing each element, we adjusted the codes, concept areas and preliminary themes, as needed.

## Results

 We identified thirteen entities, including four commercial data brokers, involved in the collection, processing and use of proprietary databases containing Canadian primary care patient data (Tables 1 and 2). Only one of the four commercial data brokers, IQVIA, was currently active in Canada. It has a proprietary database — the “IQVIA Canada EMR (AppleTree)” database^5^ — with 1.2 million Canadian patient records, mostly from primary care. IQVIA was formed when IMS Health and Quintiles, two multi-national companies, merged in 2016. At the time of the merger, IMS Health owned a Canadian primary care proprietary database, the “IMS Evidence 360 EMR Canada” database^64^ with 950 000 patients. This database was developed by IMS Brogan, a commercial data broker based in Canada and subsidiary of IMS Health since 2010. We identified a subsidiary of IQVIA, Privacy Analytics, that reported de-identifying Canadian primary care patient data. We also identified an entity that intends to become a commercial data broker, MCI Onehealth, a Canadian technology company that owns primary care clinics. In investor reports, the company states that it intends to create a proprietary database from the primary care records in its possession.^65,66^ We identified an entity – AppleTree Medical Group – that collects patient data and provides them to a commercial data broker and eight entities where employees or affiliated researchers reported using the proprietary databases.^5,64,67,68^

**Table 1:** Description of Commercial Data Brokers and Subsidiaries

**Name**	**Status**	**Country**	**Description**	**Proprietary Database(s)**
MCI Onehealth	Active	Canada	Intends to become a commercial data broker	N/A
IQVIA	Active: formed by a merger between IMS Health and Quintiles, initially called QuintilesIMS	Multi-national	Commercial data broker	IQVIA Canada EMR (AppleTree) database^3^ (1) with 1.2 million Canadian patient records (Previously called: QuintilesIMS’ Canadian Ambulatory EMR database with 1.0 million patient records from Ontario, Canada^64^)
Privacy Analytics	Active: subsidiary of IQVIA (purchased by IMS Health in 2016)	Canada	Technology company that creates de-identification technology and services	N/A
IMS Health	Not active: merged with Quintiles in 2016 to become IQVIA	Multi-national	Commercial data broker	IMS Evidence 360 EMR Canada database with 950 000 patient records^68^
IMS Brogan	Not active: subsidiary of IMS Health	Canada	Commercial data broker	IMS Evidence 360 EMR Canada database with 950 000 patients^68^

Abbreviations: EMR, electronic medical record; N/A, not available.

**Table 2:** Description of Entities Involved in the Collection, Processing or End-Use of the Proprietary Databases

**Name**	**Status**	**Country**	**Description**	**Nature of Involvement**
AppleTree Medical Group	Active	Canada	Chain of outpatient clinics	Entity runs medical facilities and a virtual care platform in Canada. It provides primary care data for the IQVIA Canada EMR (AppleTree) database^3^
Asthma Canada	Active	Canada	Non-profit patient advocacy organization	An affiliated researcher used the QuintilesIMS’ Canadian Ambulatory EMR database^64^
AstraZeneca	Active	Multi-national	Pharmaceutical company	Employees used the IMS Evidence 360 EMR Canada database^68^
McMaster University	Active	Canada	Public University	An affiliated researcher used the QuintilesIMS’ Canadian Ambulatory EMR database^64^
Medial EarlySign	Active	Multi-national	AI company	Employees used the IQVIA Canada EMR (AppleTree) database^3^
Teva Pharmaceuticals	Active	Multi-national	Pharmaceutical company	Employees used the QuintilesIMS’ Canadian Ambulatory EMR database^64^
The Lung Centre	Active	Canada	Teaching and Research Facility at a Public University	An affiliated researcher used the QuintilesIMS’ Canadian Ambulatory EMR database^64^
University of Calgary	Active	Canada	Public University	An affiliated researcher used the IMS Evidence 360 EMR Canada database^68^
University of Ottawa	Active	Canada	Public University	An affiliated researcher used the IMS Evidence 360 EMR Canada database^68^

Abbreviation: EMR, electronic medical record.

###  Documents

 We identified 25 documents from these thirteen entities that met our inclusion criteria (Supplementary file 3). The documents were published between 2013 and January 2021, and were accessed between September 5, 2018 and March 25, 2021. The intended audiences for the documents were largely data users, often the pharmaceutical industry (D1, D2, D3, D4, D9, D14, D15, and D17). Some were also addressed to shareholders (D13, D21, D22, D23, D24, and D25), and one was addressed directly to policy-makers (“Government” (D5)) (Supplementary file 3). Data brokers addressed the pharmaceutical industry to describe their data products. For example, a document on a data broker’s website, described the electronic medical record (EMR) data holdings stating, “This data is now available from IMS Brogan for the Canadian market and studies can be undertaken with the Canadian RWE [Real World Evidence] team” [D15]. Other documents also echoed this statement, informing the pharmaceutical industry that, just like researchers at academic institutions (who have access to de-identified patient data via public and non-profit data repositories), they too could access health data through various sources, including Canadian de-identified patient records.

 The intended audiences also included, at least in some cases, non-profit research organizations, governments, and academic data users. The relationships between the data brokers and these entities were often framed as collaborations or partnerships. For example, in a presentation to a non-profit health economics organization, a data broker promoted its data product by stating, “Launched in 2013 using data from 750 000 Canadian [EMRs] – partnerships with many academic institutions” [D18]. Similarly, another document stated “Federal and provincial governments also count on our solutions to serve as an extension of their teams” [D5]. These statements imply that governments, academics and data brokers can operate in synergy, and in some cases, the collaborations are key to an organization’s operations. None of the documents appeared to be directed at the public or patients.

 Thus, because the documents were largely directed at data users and shareholders, they sought to demonstrate the value of the proprietary databases. They accomplished this by describing the data holdings and providing examples of how the data can be used. However, they also constructed the value of these data more broadly by demonstrating that the creation and use of the databases provided societal benefit and entailed minimal risks. We describe the ways that the value and legitimacy of these databases are constructed and provide additional illustrative examples in Supplementary file 4.

###  Demonstrating Value: The Data Are De-identified, Patient-Level, Intimate, and Extensive

 The documents emphasized that the data are de-identified, patient-level data. A data broker’s privacy code stated: “IQVIA never has access to a patient record or prescription, which identifies the patient. The information collected does not identify any patient; it may include the age and gender of a patient” [D12]. Physician information, and that of other health professionals, however, may be identified. The document went on to state that the proprietary databases have “information collected by IQVIA concerning the diagnosis or treatment of diseases by identifiable health professionals” [D12].

 The documents claim that de-identification of patient data has important implications for consent. In a joint presentation (given by employees at a data broker and a pharmaceutical company) meant to dispel myths about EMR data, this question was posed:

 “ ***True **or **False**: Patients need to provide permission to use EMR data for research.*

*If the personal health information has been properly de-identified and the risk of re-identification tested, then this is* ***False*** *. Physician permission is required”* [D3].

 The documents explain that once data are properly de-identified, patient consent is no longer required. Instead, data brokers can ask the patient’s physician for consent. To support this claim, the presentation refers to a document co-authored by the Information and Privacy Commissioner from Ontario called “Dispelling the Myths Surrounding De-identification: Anonymization Remains a Strong Tool for Protecting Privacy”^69^ [D3], which implicitly suggests regulatory authority support. The documents also emphasized that the data are still patient-level data, not aggregated data (ie, multiple patients’ information combined together), suggesting greater analytic utility and value.

 Across the sample, the documents from data brokers described the many intimate details contained in the records – diagnoses, lab test results, time off work, smoking status, specialist referrals, and in some cases highly sensitive information like “Ethnicity/SES [socio-economic status]” and “Patient Portal Outreach QOL [quality of life] Surveys” [D4]. This contrasts to other data sources historically accessed by the pharmaceutical industry — prescription, claims or hospital databases — which typically only capture main diagnoses and prescriptions. A document on a data broker’s website describing how EMR data can be used for “research and analysis, better health metrics and product innovation” [D6] explained, “Before working with Privacy Analytics, IMS Brogan had access to prescription and claims data, which had much less patient identifying information in it, but as a result, lacked the rich analytic value of EMR data.” This degree of information gave the databases value because it enabled “highly detailed performance analytics reporting and research” [D6]. The documents also emphasized the numbers of patients in the proprietary databases – “drawn from thousands of physicians” [D4], “1.2 million individuals from AppleTree Medical Group” [D10],* “*one of the largest de-identified primary care databases in Canada” [D13]. These statements indicate that database size is a major factor determining its utility. The large databases “represent the Canadian population as a whole” [D15] and allow “statistically robust” analyses [D3].

 Documents also constructed the databases as valuable because the records are from primary care. Primary care records contain data related to the wholepatient pathway over time, in some cases containing records dating back decades. For example, a poster presentation given by an employee of a data broker explained, “Furthermore, the power of longitudinal EMR patient data is that it permits a greater understanding of the relationship between testing, diagnosis, treatment and outcomes, in the investigation of many disease states beyond gonorrhea” [D17]. These data become even more valuable when linked to data from other sources (eg, hospitals, clinical trials) at the patient-level because of the additional information. A document on a data broker’s website stated, “Encryption methodologies allow for de-identification, blending and linking data across various datasets, illustrating the full patient journey” [D15]. As a result, analysts can gain a deeper understanding of the impact of different interventions than a database with less information and a shorter time window.

###  Demonstrating Value: The Data Generate Clinical Evidence 

 The documents positioned the proprietary databases as valuable because they contain, not just data, but clinical evidence. This “real world evidence” [D15], defined as “patient-level data not collected in randomized controlled trials” [D2] provides useful information about “performance in the real world” [D9]. Real-world evidence comes from a variety of sources, including observational studies, patient registries, wearables and EMR data.

 The real-world evidence from EMR databases was characterized as particularly valuable. Slides from a joint presentation given by employees from a pharmaceutical company and a data broker include the statement, “EMR data has been used by NICE [National Institute for Health and Care Excellence] and other HTA [health technology assessment] bodies in Europe for a very long time and is considered the gold standard for Real World Evidence research” [D3]. NICE is an independent public body of the Department of Health in England with a role that includes assessing an intervention’s clinical effectiveness. NICE’s work helps to inform governmental drug approvals. Describing EMR data as “the gold standard” in this context invokes the notion of the accepted benchmark against which other data sources are judged.

 To further demonstrate the value of the proprietary databases, the documents compared the clinical evidence generated by the proprietary databases to evidence by randomized controlled trials. They characterized data in the proprietary databases as “the new currency in healthcare” [D8, D4], implying that the data from randomized controlled trials, the old currency, is no longer sufficient. Instead, the documents argued, real-world evidence is needed to fill the gaps and “complement” [D15] clinical trial data. Although data from randomized controlled trials can show that a treatment works, only real-world evidence can show how much of an impact it would have for a particular jurisdiction. Additionally, the documents claimed that data in the proprietary databases better reflects what actually happens in the real-world than trials do, because it includes the complete patient population and provides a larger volume of information.

 Documents provided examples of how pharmaceutical companies could use this new form of clinical evidence to help gain regulatory approval (“market access” [D15]) and to demonstrate value, the cost effectiveness of a therapeutic product. For example, documents described how to demonstrate “unmet need” – a situation where current management strategies do not alleviate the morbidity and mortality for individuals with a particular health condition. A document authored by a pharmaceutical company reported on one such study: “The objectives of this study were to understand a gout population in terms of demographics, clinical characteristics, healthcare utilization and costs versus a gout-free population” [D14]. This allows companies to “quantify the impact of a disease on Canadians” [D9] and to “facilitate discussions with payer and policy-makers” [D9]. Similarly, a White Paper on a data broker’s website titled “Understanding Diseases and Treatments with Canadian Real-world Evidence for Successful Market Access” stated that pharmaceutical companies could use the databases to “supplement evidence package for CDR [Canadian Agency for Drugs and Technologies in Health (CADTH) Common Drug Review] and PCPA [pan-Canadian Pharmaceutical Alliance]” [D9], two organizations which contribute to reimbursement decision-making by provincial public drug insurance plans.

###  Demonstrating Value: The Data Have Other Uses That Benefit the Pharmaceutical Industry 

 Documents also described additional uses for the proprietary databases, such as marketing and market research. According to the documents, the databases have broad marketing uses that “Can Improve Competitive Position Throughout a Brand’s Life Cycle” [D15]. A life cycle of a drug starts with initial development of the drug, and lasts through to the stage where the drug is off-patent and in competition with generic versions of the same drug. For example, the documents suggest that pharmaceutical companies could use the databases to assist with market research (eg, understanding how to market a product) and competitive research (ie, research on business competitors’ products) [D7, D9], “to differentiate and position a brand” [D15] and “to build credibility and raise awareness” through publishing “in journals and presenting at conferences” [D9].

 The databases also described how information in the databases (eg, “diagnosis” [D3, D4], “first Rx [prescription] and refills” [D4], “persistence and compliance” [D4, D7], and “dose escalation” [D9]) could be used to better understand “physician behaviour” [D12] or “prescribing behaviour” [D4]. In a joint presentation given at a pharmaceutical industry conference [D3], two Directors of a data broker and pharmaceutical company, respectively, described how the data can be used to understand how prescribers select medications for diabetes and the patient characteristics that are accounted for, including disease severity and co-morbidities, for example [D3]. The Directors concluded their presentation explaining, “The evidence is used for access purposes and for better understanding the decision points by physicians.” Thus, in understanding physician “behaviours” related to prescribing decisions, pharmaceutical companies could identify points of intervention, which documents characterized as “education.” For example, one document, a data broker’s privacy code, described:

 “*Pharmaceutical companies use the information to educate prescribers and to better understand their information needs with respect to effective and cost-efficient prescribing practices and new products and therapies”* [D12].

 Information on physician behaviour, therefore, assists pharmaceutical companies in ensuring that the information is tailored and relevant to a physician’s particular decision-making context and personal characteristics. As the databases contained physicians’ personal information, such as “age, gender, office and preferred mailing address” [D12], the data could be used to target individual physicians. The use of the term “prescriber” rather than physician may also indicate that pharmaceutical companies are targeting other healthcare professionals with prescribing privileges (such as nurse practitioners), in addition to physicians.

###  Claiming Social Legitimacy: Data Uses Benefit Society

 The documents positioned the creation and use of proprietary databases as providing societal benefit. Documents claimed that data uses will improve “health care decision-making” [D2], as well as “patient outcomes” [D13] and provide better “healthcare overall” [D6]. The documents implied that the benefits were not just for the data users, but to all of society. These messages constructed data brokers’ business activities – the collection and commercialization of patient data – as socially legitimate (ie, beneficial, ethical, and acceptable). Additionally, these benefits, according to the documents, came from the work of data users in all sectors — the pharmaceutical industry, academia, and government — all providing society with “better products and treatments” [D6]. A webpage on a data broker’s website directed at federal and provincial governments explained,

 “*Over the years, IQVIA (Canada) has worked with countless health professionals, academic institutions, pharmaceutical manufacturers and governments to provide evidence-based information to support advances in healthcare. The unique value of that information has been unlocked by these stakeholders — to increase public awareness, help shape public policy and improve the well-being of millions of Canadians*” [D5].

 The documents implied that over time, data uses by all data users, lead to better health for society. These benefits, however, could not be realized until the companies “unlocked” the information and created the proprietary databases.

###  Claiming Social Legitimacy: Privacy Loss Is not a Risk

 Another aspect of social legitimacy is addressing and mitigating the perception of risks. Accordingly, the documents identified privacy loss from re-identification of an individual as a potential risk, while claiming that data brokers have solved the problem through technical means. Documents contained diagrams and descriptions of the privacy software imbedded in the processes of collecting and storing data. A document on a data broker’s website, explained, “Through a wide variety of privacy-enhancing technologies and safeguards, QuintilesIMS protects individual privacy, while managing information to drive healthcare forward” [D9]. According to the documents, these proprietary privacy software technologies met or exceeded “Canadian privacy requirements” [D9] and ensured that “patient privacy are never compromised” [D8]. These statements implied that the risk of privacy loss is not just mitigated, but eliminated.

 The documents, however, may contain health professional identifiers. According to a data broker’s document titled “Code for the Management of Protected Information Respecting Health Professionals” [D11], although the information does not “identify any patient,”

 “*It may also include Protected Information about the health professional in the context of his or her practice: the name or other identifier, age, gender, office and mailing address, hospital affiliations, specialization and year of qualification, and information concerning diseases diagnosed and treated by them and drugs dispensed under their prescriptions”* [D11].

 The data broker seeks the “written agreement” [D11] of physicians to collect and use this identified information. The documents do not further describe why identified physician information is collected.

 One document on the website of a data broker subsidiary, describing how a data broker gained access to primary care records in Canada, delved deeper into the risk of re-identification of patient information. The document stated that the data broker wanted to gain access to up to 5 million patient EMRs from the province of Ontario. These records would provide the company with data that was “much richer than what [the data broker] had access to before” [D6]. The document stated, however, that this meant that the data contained more patient “identifying information.” Further the document acknowledged that the proprietary tools and technical approaches may not completely eliminate the risk of privacy loss, because of the need to maintain data utility. For data to have “rich analytic value” [D6], it needs to maintain detailed information on large numbers of patients. This type of data presents risks to privacy — more patients and more datapoints increases the chances that patients could be identified in datasets. The document suggested, therefore, that some compromise is needed.

 “*The amount of change made by de-identification to data utility is important and very context driven. All stakeholders need to provide input on what is most important to them, be it data utility or privacy. It’s not easy to balance the needs of everyone involved, but good communication and a commitment to producing useful data that keeps the risk of re-identification low is all you really need to get started. It’s not an easy negotiation* — *and it may be iterative* — *but it is an important negotiation to have” *[D6].

 This document, therefore, described the trade-offs between data utility and privacy, and put these considerations on equal footing. The document acknowledged there is no simple solution to resolve the conflict and leaves the decision to the stakeholders. Although the same document stated “privacy will never be compromised” this statement implied that it could be, at least to some degree, if stakeholders determined that benefits outweighed the risk of harm.

 Public annual reports from IQVIA (the only publicly traded data broker in our analysis) to shareholders also discuss the privacy risks inherent to the data collections. While re-iterating that the company has a “process and technologies to manage privacy” [D23], the documents describe how privacy concerns from privacy advocates and regulators concerns may affect access to data and the company’s “profitability” [D25], explaining,

 “*There is ongoing concern from privacy advocates, regulators and others regarding data protection and privacy issues, and the number of jurisdictions with Privacy Laws has been increasing. Also, there are ongoing public policy discussions regarding whether the standards for de-identified, anonymous or pseudonymized health information are sufficient, and the risk of re-identification sufficiently small, to adequately protect patient privacy. These discussions may lead to further restrictions on the use of such information. There can be no assurance that these initiatives or future initiatives will not adversely affect our ability to access and use data or to develop or market current or future services”* [D22].

 The documents, therefore, indicate the need for data brokers and other stakeholders to address the perceived privacy risks associated with proprietary databases as a matter of viability for the industry. Without addressing the benefits and legitimacy of these activities, documents reflect the risk that changes to privacy laws and regulation may limit data brokers’ access to the data and restrict data uses.

## Discussion

 Our content analysis provides insight into the creation and use of the proprietary databases containing Canadian primary care records and the ways these databases are constructed as valuable and socially legitimate. The documents described the databases as valuable to the pharmaceutical industry, governments and academics because they contain extensive, patient-level, de-identified information on millions of Canadians and can be used to generate real-world evidence – “data regarding the usage, or the potential benefits or risks, of a drug derived from sources other than traditional clinical trials.”^70,71^ The databases can also assist pharmaceutical companies with marketing their products and understanding physician behaviour. The documents constructed the value of the proprietary databases more broadly by claiming they improved health for patients, while also addressing risks to privacy.

 The documents positioned the proprietary databases as becoming increasingly valuable to the pharmaceutical industry, in part because of requests from regulators and funders for real-world evidence. In 2016, the US mandated in the 21^st^ Century Cures Act that the Food and Drug Administration (FDA) develop a program to use real-world evidence to support new drug approvals.^70,71^ One important source of real-world evidence according to the FDA, is data from EMRs.^71^ Although real-world evidence for effectiveness of an intervention is at higher risk of bias from lack of randomization,^72,73^ it is far less costly to gather; includes a broader range of patients; and allows regulators to assess drug efficacy more rapidly in emergency situations and for rare conditions, where a trial may not be feasible.^74-80^ Following the FDA, regulators in Canada and other jurisdictions are also starting to incorporate real-world evidence into regulatory and funding approval processes.^75,81-83^

 Some documents indicated that the messages promoting data value and asserting patient privacy exhibit trade-offs, which suggests these considerations may be in tension. For the databases to be useful to data customers, they must contain large amounts of patient-level, detailed health information, ideally linked across multiple data sources.^7,84-86^ Research shows that with these kinds of databases, the risk of privacy loss (and exposure of sensitive information) is ever present and often unpredictable.^7,26-28,87^ The documents also interpreted privacy narrowly, focusing solely on loss of anonymity (re-identification of an individual within a dataset). Privacy risks, however, can be conceptualized much more broadly and beyond the loss of anonymity for individuals. The Information and Privacy Commissioner of Ontario discusses these ethical issues in a recent article titled “Ripe for public debate: Legal and ethical issues around de-identified data.” She describes how de-identified data can be used to make inferences about groups that share similar characteristics and how this can cause “stigmatization and discrimination, unfair distribution of services or benefits, loss of jobs, or denial of insurance coverage.”^23^

 Our work also indicates that the creation of the proprietary databases — containing health professionals’ identified information — may enable more effective drug promotion to prescribers. The documents positioned the proprietary databases as valuable, in part, because they contain detailed information on physician decision-making and prescribing behavior. One document describes how the information might be used: to optimize outreach to prescribers (physicians, nurse practitioners). Work by other researchers demonstrates how information about physician behaviour can enhance drug promotion and have problematic consequences.^3,37,39^ In a recent publication, authors Mulinari and Ozieranski describe how additional detailed information on physician behaviour in the Open Payments Database, made public through the Physician Payments Sunshine Act,^88^ allowed pharmaceutical companies to “sharpen their marketing tools.”^39^ As the database contained a record of all pharmaceutical industry in-kind and cash payments to physicians, it helped pharmaceutical companies identify new physician targets — in particular, those who were “commercially the most relevant to the company” — for promotional activities. Similarly, the pharmaceutical industry may find ways to use the extensive information on physician behaviour in the proprietary databases to improve physician surveillance and marketing. Problematically, studies repeatedly demonstrate that these type of promotional activities influence medical practice by leading physicians to prescribe more drugs, more expensive drugs and to make less appropriate prescribing choices.^89^

###  Next Steps

 Our analysis indicates the need for democratic processes to enable important secondary uses of health data — such as determining whether a drug should receive regulatory approval or monitoring for after-market harms — while addressing risks from the creation and use of proprietary databases. Solutions should allow data to be used for the public good (substantial public benefit and clear scientific value^42,63^), while addressing risks, like loss of anonymity, surveillance, discrimination and problematic drug promotion, as well as ethical concerns such as who should have access to and control over data.^90-92^ Many researchers and theorists have used ethical principles and public values to create frameworks and guidance that address these issues.^42,43,91,93-96^ Their recommendations include policies that require data to be held by trusted entities, like public organizations or non-profit community research groups; diverse governance, including patient stakeholders; data sovereignty (data ownership and control) for marginalized communities; appropriate consent mechanisms; transparency for all process and decisions; and external regulatory and ethical oversight. To date in Canada, however, governments have been slow to create public mechanisms and infrastructure to provide access to health data.^63^

###  Strengths and Weaknesses

 This study employed a critical qualitative methodology which understands that texts are value-laden and situated within a specific social context and power structures.^54,56^ These methods are interpretive. Thus, our analysis represents just one possible reading of these texts, which is grounded in documentary evidence from a variety of sources and perspectives including data brokers, consulting companies and data users (eg, academics, disease organizations and pharmaceutical companies). We were limited, however, to publicly available documents and thus, have likely captured just a fraction of published accounts of the collection and secondary uses of primary healthcare data. For example, internal company documents may have provided more insight into how the value of these proprietary datasets were constructed and the trade-offs between privacy and data utility. For auditability, we included appendices with our search strategies and further supporting evidence of our interpretations. However, due to the tailored nature of web-based searching, it is unlikely that these searches are fully replicable. Our study focused on the Canadian context and, although data brokers collect primary care health data from many countries around the world, differing political, legal, and social contexts may affect the applicability of our findings.^4^ Thus, our analysis should serve as a starting point to prompt discussion and further inquiry.

## Conclusion

 Data brokers have proprietary databases containing millions of de-identified Canadian primary care records. Documents from data brokers, and other entities involved in the collection and use of these records, constructed the proprietary databases as valuable, particularly to the pharmaceutical industry. The data could be used to demonstrate safety and efficacy to regulators and funders; assist with marketing; and provide insight into prescriber behaviour. The documents constructed the value of these data more broadly by claiming to improve health for patients, while also addressing risks to privacy. The collection and use of large amounts of intimate patient-level information, however, may present risks to privacy; enable surveillance and targeting of patients who share similar characteristics; and contribute to problematic drug promotion. Solutions could include public data repositories, external regulatory oversight, transparency for all data uses and appropriate consent mechanisms.

## Acknowledgments

 The authors would like to thank the Women’s College Hospital Peer Support Writing Group for reading a draft of this article and providing feedback and Susan Hum for editing a final draft.

## Ethical issues

 This study did not require research ethics approval as it used publicly available documents.

## Competing interests

 Authors declare that they have no competing interests.

## Authors’ contributions

 Conceptualization: Sheryl Spithoff and Quinn Grundy.

 Formal analysis: Sheryl Spithoff and Quinn Grundy.

 Funding acquisition: Sheryl Spithoff and Quinn Grundy.

 Investigation: Sheryl Spithoff and Quinn Grundy.

 Methodology: Sheryl Spithoff and Quinn Grundy.

 Writing–original draft: Sheryl Spithoff.

 Writing–review & editing: Quinn Grundy.

## Funding

 SS received funding from a New Investigator award from the Department of Family and Community Medicine, University of Toronto. This work was supported in part by a grant from the Social Sciences and Humanities Council of Canada (SSHRC) (430-2021-00207). The study funder had no role in data collection, interpretation or reporting.

## Supplementary files


Supplementary file 1. Standards for Reporting Qualitative Research.

Supplementary file 2. Identification of Entities and Documents.

Supplementary file 3. Characteristics of the Documents Included in the Analysis.

Supplementary file 4. Quotations.
