# Supplementary material for: Commercializing Personal Health Information: A Critical Qualitative Content Analysis of Documents Describing Proprietary Primary Care Databases in Canada
Source: Int J Health Policy Manag. 2023 May 2;12:6938. doi: 10.34172/ijhpm.2023.6938 (PMC10461871; doi:10.34172/ijhpm.2023.6938)
Supplement: Supplementary file 2 — Identification of Entities and Documents. [file ijhpm-12-6938-s002.pdf]

Article title: Commercializing Personal Health Information: A Critical Qualitative Content Analysis of Documents Describing Proprietary Primary Care Databases in Canada

Journal name: International Journal of Health Policy and Management (IJHPM)

Authors' information: Sheryl Spithoff[1,2,3]*, Quinn Grundy[4]

[1]Department of Family and Community Medicine, University of Toronto, Toronto, ON, Canada.

[2]Department of Family and Community Medicine, Women's College Hospital, Toronto, ON, Canada.

[3]Women's College Research Institute, Women's College Hospital, Toronto, ON, Canada.

[4]Lawrence S. Bloomberg Faculty of Nursing, University of Toronto, Toronto, ON, Canada.

(*Corresponding author: Sheryl.spithoff@wchospital.ca)

Supplementary file 2. Identification of Entities and Documents

Step 1: Identifying entities (iterative search)

Table 1. Identifying entities, initial search*

Mechanism	Name	Search	Entities identified
Search engine	Google (first 100 results)	("Anonymize" OR "de-identify") AND "Canada" AND ("primary care" OR "electronic medical records" OR "patient data") AND (company OR companies OR corporation)<br><br>"Canada" AND ("real world evidence" OR "real world data")	IQVIA<br>IMS Brogan<br>IMS Health<br>Quintiles IMS<br>Privacy Analytics<br>MCI Onehealth<br>AstraZeneca

*conducted multiple times between September 2018 and March 2021

Table 2. Iterative identification of entities*

Mechanism	Name	Search	Entities identified
Search engine	Google (first 100 results)	["Company name"] AND "primary care" AND "Canada" AND (Anonymize OR de-identify)	None additional
Database	Google Scholar	["Company name"] AND "primary care" AND "Canada"	Appletree Medical Group<br>Medial EarlySign

*conducted multiple times between September 2018 and March 2021

Step 2: Identifying documents and additional entities (iterative search)

Table 3. Identifying documents and additional entities*

Mechanism	Name	Search	Documents and additional entities
Search engine	Google (first 100 results)	[Company name] AND ("primary care" OR " electronic medical records" OR "EMR" OR "patient data" OR "real world data" OR "real world evidence") AND (Canada OR Canadian)<br><br>["Commercial database name"] AND Canada<br><br>["parts of Commercial database name"] AND Canada	D1, D2, D3, D6, D18
Database	Google Scholar<br>Pubmed	[Commercial database name]	D10, D11, D14, D15
Website search	Website and Google site search	Informal searching of company website<br><br>Formal search of website using Google site search function<br>site: [Company domain name] AND ("primary care" OR " electronic medical records" OR "EMR" OR "patient data" OR "real world data" OR "real world evidence" OR "RWD" OR "RWE")	D5, D7, D8, D9, D12, D13, D16, D21, D22, D23, D24, D25
Bibliography on data broker's website	www.rwebibliography.com/	Search of IQVIA Real-World Insights Bibliography using term "Canada" from 2013 to 2021(all 43 results) most were not accessible nor available online	D17, D19, D20<br><br>Asthma Canada

*conducted multiple times between September 2018 and March 2021
