# Supplementary material for: Commercializing Personal Health Information: A Critical Qualitative Content Analysis of Documents Describing Proprietary Primary Care Databases in Canada
Source: Int J Health Policy Manag. 2023 May 2;12:6938. doi: 10.34172/ijhpm.2023.6938 (PMC10461871; doi:10.34172/ijhpm.2023.6938)
Supplement: Supplementary file 3 — Characteristics of the Documents Included in the Analysis. [file ijhpm-12-6938-s003.pdf]

Article title: Commercializing Personal Health Information: A Critical Qualitative Content Analysis of Documents Describing Proprietary Primary Care Databases in Canada

Journal name: International Journal of Health Policy and Management (IJHPM)

Authors' information: Sheryl Spithoff[1,2,3]*, Quinn Grundy[4]

[1]Department of Family and Community Medicine, University of Toronto, Toronto, ON, Canada.

[2]Department of Family and Community Medicine, Women's College Hospital, Toronto, ON, Canada.

[3]Women's College Research Institute, Women's College Hospital, Toronto, ON, Canada.

[4]Lawrence S. Bloomberg Faculty of Nursing, University of Toronto, Toronto, ON, Canada.

(*Corresponding author: Sheryl.spithoff@wchospital.ca)

Supplementary file 3. Characteristics of the Documents Included in the Analysis

	Document title	Sampled entity	Author	Date created	Type of document	Audience	Document link	Citation
D1	Assessment of a Canadian primary care electronic medical records database for use in observational studies	IMS Brogan AstraZeneca	S Frise (AstraZeneca Canada Inc), K Reidel (IMS Brogan Canada), JE Tarride (AstraZeneca Canada Inc), N Corner (IMS Brogan Canada, A Dziarmaga (AstraZeneca Canada Inc.)	2013	Abstract published in industry journal and document on company website	Pharmaceutical industry	https://www.valueinhealthjournal.com/article/S1098-3015(13)03503-1/pdf https://web.archive.org/web/20160504033124/http://imsbrogancapabilities.com/pdf/rwe/assessment-of-a-primary-care-database.pdf	Frise, S., K. Reidel, J. E. Tarride, N. Corner, and A. Dziarmaga. "Assessment of a Canadian Primary Care Electronic Medical Record Database for Use in Observational Studies." *Value in Health* 16, no. 7 (November 1, 2013): A582. https://doi.org/10.1016/j.jval.2013.08.1598.
D2	Real World Examples of Real World Evidence	IQVIA	Brad Millson (IQVIA)	2018	Presentation at therapeutics conference	Pharmaceutical industry	https://web.archive.org/web/20201223024608/https://www.capt-actp.ca/wp-content/uploads/2018/11/session-1-slides.pdf	Millson, Brad. "Real World Examples of Real World Evidence." Canadian Association for Population Therapeutics (CAPT) Conference, October 2018. https://www.capt-actp.ca/wp-content/uploads/2018/11/session-1-slides.pdf.

D3	Delivering Real World Evidence from Electronic Medical Records in Canada "Let's Get Real!"	IMS Health<br>IMS Brogan AstraZeneca	Neil Corner (IMS Brogan) and Alison Dziarmaga (Astra Zeneca Canada Inc.)	2015	Presentation at pharmaceutical industry conference	Pharmaceutical industry	https://web.archive.org/web/20211102142221/https://docplayer.net/1247214-Delivering-real-world-evidence-canada-let-s-get-real.html	Corner, Neil, and Alison Dziarmaga. "Delivering Real World Evidence. Canada Let's Get Real!" Presented at the Canadian Pharma Market Research Conference, 2015. https://docplayer.net/1247214-Delivering-real-world-evidence-canada-let-s-get-real.html.
D4	Electronic Medical Records (EMR)<br>The Most Comprehensive Source of Unique Real-World Evidence (RWE) Insights on Patient-Level Data in Canada	Quintiles IMS (IQVIA)	Anonymous	2017	Document on company website	Pharmaceutical industry	https://imsbrogancapabilities.com/pdf/emr-data.pdf.	QuintilesIMS. "Electronic Medical Records (EMR) The Most Comprehensive Source of Unique Real-World Evidence (RWE) Insights on Patient-Level Data in Canada," 2017. https://imsbrogancapabilities.com/pdf/emr-data.pdf.
D5	Government	IQVIA	Anonymous	No date	Document on company website	Federal and provincial governments	http://hiis.imsbrogan.com/en/government.html	IQVIA. "Government." IQVIA. Accessed September 5, 2018. http://hiis.imsbrogan.com/en/government.html.
D6	IMS Health: Unlocking the Value of EMR Data for Advanced Research and Analysis, Better Health Metrics, and Product Innovation	Privacy Analytics, IMS Health, IMS Brogan	Anonymous	2017	Document on company website	Not clear	https://web.archive.org/web/20210216165758/https://privacy-analytics.com/wp-content/uploads/dlm_uploads/2020/06/IMS-Brogan-Case-Study.pdf<br><br>https://privacy-analytics.com/wp-content/uploads/dlm_uploads/2020/06/IMS-Brogan-Case-Study.pdf	Privacy Analytics. "IMS Health: Unlocking the Value of EMR Data for Advanced Research and Analysis, Better Health Metrics, and Product Innovation." QuintilesIMS, 2017. https://privacy-analytics.com/wp-content/uploads/dlm_uploads/2020/06/IMS-Brogan-Case-Study.pdf. https://web.archive.org/web/20210216165758/https://privacy-analytics.com/wp-content/uploads/dlm_uploads/2020/06/IMS-Brogan-Case-Study.pdf.
D7	IMS Health Real-World Data<br>A straightforward way to get real-world data	IMS Health	Anonymous	2015	Document on company website	Data customers	https://web.archive.org/web/20160910085222/http://imsbrogancapabilities.com/pdf/real-world-data-fact-sheet.pdf	IMS Health. "A Straightforward Way to Get Real-World Data," 2015. https://web.archive.org/web/20160910085222/http://imsbrogancapabilities.com/pdf/real-world-data-fact-sheet.pdf.
D8	REAL-WORLD EVIDENCE Canadian Real-World Data from De-Identified EMR	IQVIA	Anonymous	2017	Document on company website	Data customers	https://web.archive.org/web/20180425121650/imsbrogancapabilities.com/en/health-access/real-world-evidence.html	IQVIA. "REAL-WORLD EVIDENCE Canadian Real-World Data from De-Identified EMR." https://web.archive.org/web/20180425121650/imsbrogancapabilities.com/en/health-access/real-world-evidence.html. Accessed November 28, 2018. https://web.archive.org/web/20180425121650/imsbrogancapabilities.com/en/health-access/real-world-evidence.html.
D9	Understanding Diseases and Treatments with Canadian Real-world Evidence	IMS Health<br>IMS Brogan (2014)	Anonymous	2014	Document on company website	Pharmaceutical industry	2014 version<br>https://web.archive.org/web/20160503151919/http://imsbrogancap	IMS Brogan. "Understanding Diseases and Treatments with Canadian Real-World Evidence - PDF Free Download," 2014. https://web.archive.org/web/20160503151919/http://i

	Real-World Evidence for Successful Market Access: White paper						abilities.com/pdf/healthcare-real-world-evidence-3.pdf	msbrogancapabilities.com/pdf/healthcare-real-world-evidence-3.pdf.
D10	Prediction of progression from pre-diabetes to diabetes: Development and validation of a machine learning model	Medial EarlySign	Avivit Cahn (Medial EarlySign), Avi Shoshan, Tal Sagiv (Medial EarlySign),, Rachel Yesharim (Medial EarlySign),, Ran Goshen, Varda Shalev, Itamar Raz (Medial EarlySign),	2019	Journal article	Medical audience	https://doi.org/10.1002/dmrr.3252	Cahn, Avivit, Avi Shoshan, Tal Sagiv, Rachel Yesharim, Ran Goshen, Varda Shalev, and Itamar Raz. "Prediction of Progression from Pre-Diabetes to Diabetes: Development and Validation of a Machine Learning Model." *Diabetes/Metabolism Research and Reviews* 36, no. 2 (2020): e3252. https://doi.org/10.1002/dmrr.3252.
D11	Partner With Us	Appletree Medical Group	Anonymous	2020	Company webpage	Organizations that may be interested in partnering with a medical clinic	https://web.archive.org/web/20201223024055/https://appletreemedicalgroup.com/partner-with-us/	Appletree Medical Group. "Partner With Us | Employers & Pharmacies | Appletree Medical." Accessed December 22, 2020. https://appletreemedicalgroup.com/partner-with-us/.
D12	Code for the Management of Protected Information Respecting Health Professionals (Excluding Quebec)	IQVIA	Anonymous	2012, 2015	Document on company website	Health professionals and data customers	https://web.archive.org/web/20201127210228/https://www.iqvia.com/-/media/iqvia/pdfs/canada/canada-location-site/privacy-code-canada-en.pdf?la=en&hash=1EEAC2C082EC9242F478E4539A77E9BC&_=1606510824294	IQVIA. "CODE FOR THE MANAGEMENT OF PROTECTED INFORMATION RESPECTING HEALTH PROFESSIONALS (EXCLUDING QUEBEC)." IQVIA, July 2015. https://web.archive.org/web/20201127210228/https://www.iqvia.com/-/media/iqvia/pdfs/canada/canada-location-site/privacy-code-canada-en.pdf?la=en&hash=1EEAC2C082EC9242F478E4539A77E9BC&_=1606510824294.
D13	MCI Onehealth: Empowering patients and doctors with advanced technologies to increase access, improve quality, and reduce the costs of healthcare	MCI Oneheatlh.	Anonymous	2020	Document on company website	Investors	https://web.archive.org/web/20210209182923/https://investor.mcionehealth.com/static-files/78f6ac37-8913-44a6-852c-c9ef35a467da	MCI Onehealth. "MCI Onehealth: Empowering Patients and Doctors with Advanced Technologies to Increase Access, Improve Quality, and Reduce the Costs of Healthcare." December 17, 2020. https://web.archive.org/web/20210209182923/https://investor.mcionehealth.com/static-files/78f6ac37-8913-44a6-852c-c9ef35a467da.
D14	The Burden of Gout in a Canadian Primary Care Population	AstraZeneca	D.M. Williams, C Cowan, A Gendron, J Goodfield, D Oraichi, A Fischer, R Borrelli, N Liu, A Dziarmaga	2015	Journal article	Pharmaceutical industry	https://www.valueinhealthjournal.com/article/S1098-3015(15)01656-3/abstract	Williams, D. M., C. Cowan, A. Gendron, J. Goodfield, D. Oraichi, A. Fischer, R. Borrelli, N. Liu, and A. Dziarmaga. "The Burden of Gout in a Canadian Primary Care Population." *Value in Health* 18, no. 3 (May 1, 2015): A274. https://doi.org/10.1016/j.jval.2015.03.1599.
D15	Real World Evidence for the Canadian Market	IMS Health<br>IMS Brogan	Anonymous	2013	Document on company website	Pharmaceutical industry	https://web.archive.org/web/20160504033101/http://imsbrogancapabilities.com/pdf/healthcare-real-world-evidence-1.pdf	IMS Brogan. "Real World Evidence for the Canadian Market," 2013. https://web.archive.org/web/20160504033101/http://imsbrogancapabilities.com/pdf/healthcare-real-world-evidence-1.pdf.

D16	Severe, eosinophilic asthma in primary care in Canada: a longitudinal study of the clinical burden and economic impact based on linked electronic medical record data	AstraZeneca<br>University of Ottawa<br>University of Calgary	Don Husereau (University of Ottawa), Jason Goodfield (IQVIA), Richard Leigh (University of Calgary), Richard Borrelli, Michel Cloutier, and Alain Gendron (University of Montreal; AstraZeneca Canada Inc.)	2017	Journal article	Medical audience	https://aacijournal.biomedcentral.com/articles/10.1186/s13223-018-0241-1	Husereau, Don, Jason Goodfield, Richard Leigh, Richard Borrelli, Michel Cloutier, and Alain Gendron. "Severe, Eosinophilic Asthma in Primary Care in Canada: A Longitudinal Study of the Clinical Burden and Economic Impact Based on Linked Electronic Medical Record Data." *Allergy, Asthma & Clinical Immunology* 14, no. 1 (April 24, 2018): 15. https://doi.org/10.1186/s13223-018-0241-1.
D17	Using Electronic Medical Records to Better Understand the Relationship Between Testing, Diagnosis and Treatment of Gonorrhea in Ontario	IMS Health<br>IMS Brogan	Allan Gillman (IMS Brogan)	2016	Poster presentation at pharmaceutical industry Conference	Pharmaceutical industry	https://web.archive.org/web/20160503160956/http://imsbrogancapabilities.com/pdf/rwe/capt-2015-conference.pdf	Gillman, Allan. "Using Electronic Medical Records to Better Understand the Relationship Between Testing, Diagnosis and Treatment of Gonorrhea in Ontario." IMS Health, May 3, 2016. https://web.archive.org/web/20160503160956/http://imsbrogancapabilities.com/pdf/rwe/capt-2015-conference.pdf.
D18	Guiding principles for the use of Real World Evidence: What questions do we need to consider?	IMS Health<br>IMS Brogan	Neil Corner (IMS Health)	2014	Presentation to a non-profit organization	Data users	https://web.archive.org/web/20210422173745/https://www.ihe.ca/advanced-search/guiding-principles-for-its-use-what-questions-do-we-need-to-consider-	Neil Corner. "Guiding Principles for the Use of Real World Evidence: What Questions Do We Need to Consider? IMS Brogan." Presented at the Institute of Health Economics, Alberta, Canada, n.d. https://www.ihe.ca/advanced-search/guiding-principles-for-its-use-what-questions-do-we-need-to-consider-.
D19	Characteristics of Patients with Mild to Severe Asthma in Canada	QuintilesIMS<br>Teva pharmaceuticals<br>McMaster University<br>The Lung Centre<br>Asthma Canada	Sebastien K Gerega (QuintilesIMS), Brad Millson (QuintilesIMS), Katia Charland (Quintiles IMS), Stephane Barakat (TEVA Pharmaceuticals), Xichun Sun, Ricardo Jimenez (TEVA Pharmaceuticals), Susan Waserman (McMaster University), J Mark FitzGerald (The Lung Centre)	2017	Abstract of a presentation at a medical conference	Medical audience	https://web.archive.org/web/20200824234658/https://asthma.ca/wp-content/uploads/2017/06/Research-Poster.pdf	Gerega, Sebastien, Brad Millson, Katia Charland, Stephanie Barakat, Xichun Sun, Ricardo Jimenez, Susan Waserman, and J Mark FitzGerald. "Characteristics of Patients with Mild to Severe Asthma in Canada (IMSQuintiles and Asthma Canada)." Presented at the Canadian Respiratory Conference (CRC), Montreal, 2017. https://web.archive.org/web/20200824234658/https://asthma.ca/wp-content/uploads/2017/06/Research-Poster.pdf.
D20	The evolving role of real-world evidence to support policy and practice	IMS Health	Neil Corner (IMS Health)	2015	Panel presentation at Health Policy conference	Data users	https://www.cadth.ca/symposium2018/evolving-role-real-world-evidence-support-policy-and-practice	Corner, Neil. "The Evolving Role of Real-World Evidence to Support Policy and Practice." Presented at the CADTH, 2015. https://www.cadth.ca/symposium2018/evolving-role-real-world-evidence-support-policy-and-practice.
D21	Annual Report 2016	Quintiles IMS	Anonymous	2016	Investor report	Investors	http://web.archive.org/web/20220916144036/https://s24.q4cdn.com/326377938/files/doc_financials/annual/iqvia/QuintilesIMS_2016_	Quintiles IMS. "2016 Annual Report." 2016. http://web.archive.org/web/20220916144036/https://s24.q4cdn.com/326377938/files/doc_financials/annual/iqvia/QuintilesIMS_2016_Annual-Report_Final-%281%29.pdf

							Annual-Report_Final-%281%29.pdf	
D22	Annual Report 2017	IQVIA	Anonymous	2017	Investor report	Investors	http://web.archive.org/web/20220916144702/https://s24.q4cdn.com/326377938/files/doc_financials/annual/iqvia/IQVIA-2017-10K-Filed-%282%29.pdf	IQVIA. “Annual report 2017” 2017. http://web.archive.org/web/20220916144702/https://s24.q4cdn.com/326377938/files/doc_financials/annual/iqvia/IQVIA-2017-10K-Filed-%282%29.pdf
D23	Annual Report 2018	IQVIA	Anonymous	2018	Investor report	Investors	http://web.archive.org/web/20220916144831/https:/s24.q4cdn.com/326377938/files/doc_financials/annual/iqvia/IQVIA-2018-10K-%28w-exhibits%29-Filed.pdf	IQVIA. “Annual report 2018” 2018. http://web.archive.org/web/20220916144831/https:/s24.q4cdn.com/326377938/files/doc_financials/annual/iqvia/IQVIA-2018-10K-%28w-exhibits%29-Filed.pdf
D24	Annual Report 2019	IQVIA	Anonymous	2019	Investor report	Investors	https://web.archive.org/web/20220909164131/https://s24.q4cdn.com/326377938/files/doc_financials/2019/annual/IQV-2019-10-K-Filed-(with-exhibits).pdf	IQVIA. “Annual report 2019” 2019. https://web.archive.org/web/20220909164131/https://s24.q4cdn.com/326377938/files/doc_financials/2019/annual/IQV-2019-10-K-Filed-(with-exhibits).pdf
D25	Annual Report 2020	IQVIA	Anonymous	2020	Investor report	Investors	https://web.archive.org/web/20220916145713/https://s24.q4cdn.com/326377938/files/doc_financials/2020/ar/IQV-2020.12.31-10K-Final-%28with-exhibits%29.pdf	IQVIA. “Annual report 2020” 2020. https://web.archive.org/web/20220916145713/https://s24.q4cdn.com/326377938/files/doc_financials/2020/ar/IQV-2020.12.31-10K-Final-%28with-exhibits%29.pdf
