# Supplementary material for: Commercializing Personal Health Information: A Critical Qualitative Content Analysis of Documents Describing Proprietary Primary Care Databases in Canada
Source: Int J Health Policy Manag. 2023 May 2;12:6938. doi: 10.34172/ijhpm.2023.6938 (PMC10461871; doi:10.34172/ijhpm.2023.6938)
Supplement: Supplementary file 4 — Quotations. [file ijhpm-12-6938-s004.pdf]

Article title: Commercializing Personal Health Information: A Critical Qualitative Content Analysis of Documents Describing Proprietary Primary Care Databases in Canada

Journal name: International Journal of Health Policy and Management (IJHPM)

Authors' information: Sheryl Spithoff[1,2,3]*, Quinn Grundy[4]

[1]Department of Family and Community Medicine, University of Toronto, Toronto, ON, Canada.

[2]Department of Family and Community Medicine, Women's College Hospital, Toronto, ON, Canada.

[3]Women's College Research Institute, Women's College Hospital, Toronto, ON, Canada.

[4]Lawrence S. Bloomberg Faculty of Nursing, University of Toronto, Toronto, ON, Canada.

(*Corresponding author: Sheryl.spithoff@wchospital.ca)

Supplementary file 4. Quotations

Themes and concepts	Description	Quotations
**Data users: pharmaceutical industry**	The intended audiences for the documents appeared to be data users, mainly pharmaceutical and medical device companies.	Not just the domain of the academic researcher anymore, data is available to the pharmaceutical industry. D3<br><br>Unique in Canada, this database represents the only commercially available EMR research database for the pharmaceutical industry. The database contains 1.1 million unique patients' anonymized electronic medical records with 7.7 million unique visits. Some records go back as far as the mid-1990s. Update frequency is available monthly or quarterly. D4<br><br>For researchers and medical manufacturers, having access to de-identified datasets that contain accurate, up-to-date patient information and are custom-built for specific analysis, is incredibly valuable. D6<br><br>These insights and execution capabilities help biotech, medical device, and pharmaceutical companies, medical researchers, government agencies, payers and other healthcare stakeholders in the development and approval of new therapies, identify unmet treatment needs and understand the safety, effectiveness and value of pharmaceutical products in improving overall health outcomes. D9<br><br>Pharmaceutical companies use the information to educate prescribers and to better understand their information needs with respect to effective and cost-efficient prescribing practices and new products and therapies; to obtain participation in clinical trials of new products; to facilitate drug warnings and recalls; and to market their products. D12<br><br>Real World Evidence for the Canadian Pharmaceutical Market. D15

		This data is now available from IMS Brogan for the Canadian market and studies can be undertaken with the Canadian RWE [Real World Evidence] team as part of the global RWE [Real World Evidence] Centre of Excellence. D15<br><br>[T]here has been recognition that RWE [Real World Evidence] has broader applications than research, and the term RWE is often interchanged with Real World Data (RWD). These broader applications go beyond the notion of public evidence, but apply the same techniques and data to enable internal business decision-making in pharma and by payers. D15<br><br>IQVIA creates intelligent connections across all aspects of healthcare through its analytics, transformative technology, big data resources and extensive domain expertise. IQVIA Connected Intelligence™ delivers powerful insights with speed and agility — enabling customers to accelerate the clinical development and commercialization of innovative medical treatments that improve healthcare outcomes for patients. With approximately 70,000 employees, we conduct operations in more than 100 countries. D25<br><br>Our clients face escalating research and development costs even as product approval success rates remain low. Speed to market is crucial but more elusive as clinical trials require highly targeted patient populations and increasingly complex designs. After launch, companies face growing demands to measure outcomes and demonstrate the real-world value of their medicines. As a result, they must produce continuous evidence to support safety, access and pricing decisions. Meanwhile, brand and commercial teams are looking to become more effective and efficient in engaging a highly complex mix of stakeholders—increasing reliance on data and technology. D21<br><br>Sales to companies in life sciences, including pharmaceutical companies, biotechnology companies, device and diagnostic companies, and consumer health companies, account for the majority of our revenues. Nearly all of the top 100 global pharmaceutical and biotechnology companies, measured by revenue, are clients, and many of these companies subscribe to reports and services in many countries. D22<br><br>Our mission-critical relationships with our life science clients consist of four important decision-making processes related to their product portfolios: Research and Development, Pre-Launch, Launch and In-Market. We continue to develop software and services applications to further deepen our level of client integration by enabling our clients to enhance and/or automate many components of these key decision-making processes. D22<br><br>The IQVIA Institute also estimates that approximately 270 new molecular entities (“NMEs”) are expected to be approved between 2021 and 2025, compared to 234 between 2016 and 2020, and 220 between 2011 and 2015. We believe that further research and development spending, combined with the continued need for cost efficiency across the healthcare landscape, will continue to create opportunities for biopharmaceutical services companies, particularly those with a global reach and broad service offerings, to help biopharmaceutical companies with their pre- and post-launch solutions development and commercialization needs. D23<br><br>The vast majority of our revenue is generated from sales to the biopharmaceutical and healthcare industries. The clients we serve in these industries are commonly subject to financial pressures, including, but not limited to, increased costs, reduced demand for their products, reductions in pricing and reimbursement for products and services, formulary approval and placement, government approval to market their products and limits on the manner by which they market their products, loss of patent exclusivity (whether due to patent expiration or as a result of a successful legal challenge) and the proliferation of or changes to regulations applicable to these industries. To the extent our clients face such

		pressures, or they change how they utilize our offerings, the demand for our services, or the prices our clients are willing to pay for those services, may decline. Any such decline could have a material adverse effect on our business, operating results and financial condition. D25
**Data users: governments and academic researchers**	Data users also included non-profit research organizations, governments and academic institutions as they were often mentioned as data users.	Design was discussed and agreed upon in advance with FDA after close, collaborative consultations. D2<br><br>CODE is a broad Collaboration aiming to connect the European Cancer Community with data in powerful ways D2<br><br>IMS Evidence 360 cohort builder. Canadian EMR in a simple tool. Available in Europe, US too… Even the FDA uses it! D3<br><br>In addition to the pharmaceutical industry, many academic institutions are working with our EMR [electronic medical record] data, including St-Michael’s, Université de Montréal, and Toronto and Alberta Universities. D4<br><br>Our customized datasets and analyses have supported many successfully completed projects using highly powered retrospective studies, validated for scientific vigour through our network of academic groups and clinical experts. Our experienced team of Principals and Engagement managers including PhD Epidemiologists, PhD Statisticians, Data scientists, can help you ask the right questions that have led to breakthrough research. D4<br><br>Federal and provincial governments also count on our solutions to serve as an extension of their teams. D5<br><br>Technology services can also provide governments with an enhanced understanding of healthcare operations, outcomes, and costs at the patient, practice, and institutional levels. D5<br><br>IQVIA (Canada) exhibited at the 7th annual Conference of the MUHC-ISAI (McGill University Health Centre's Institute for Strategic Analysis and Innovation) which was created to equip patients, clinicians, administrators and policy makers with the inspiration, models and best-in-class tools to pursue patient engagement initiatives that improve care and outcomes. D5<br><br>These insights and execution capabilities help biotech, medical device, and pharmaceutical companies, medical researchers, government agencies, payers and other healthcare stakeholders in the development and approval of new therapies, identify unmet treatment needs and understand the safety, effectiveness and value of pharmaceutical products in improving overall health outcomes. D9<br><br>Federal and provincial government departments and agencies use the information for educational purposes; to determine adherence to applicable guidelines; to identify prescribing patterns; and generally to create healthcare policy and improve the management of healthcare resources. D12<br><br>A significant opportunity exists to collaborate with other medical institutions and leading organizations to further assemble valuable de-identified databases; unlocking both clinical and commercial potential. D13<br><br>Partnerships with many academic institutions. D18

		Other clients include payers, government and regulatory agencies, providers, pharmaceutical distributors, and pharmacies. Our client base is broad in scope and enables us to avoid dependence on any single client. No single client accounted for 10% or more of our total company revenues in 2020, 2019, or 2018. As of December 31, 2020 the largest client based on its percentage of total company revenue contributed approximately 5%. D22<br><br>We help our global customers across payers, providers, governments, and biopharmaceutical companies to answer critical questions about healthcare interventions related to safety, effectiveness, and value. D24
**Demonstrating value**	The patient data are de-identified	Permission granted for secondary use of data by 152 physicians with support from 345 other Health Care Professionals (HCP). D1<br><br>**True or False**: Patients need to provide permission to use EMR data for research.<br>If the personal health information has been properly de-identified and the risk of re-identification tested then this is **FALSE**. Physician permission is required. D3<br><br>IMS Brogan has addressed this need with a comprehensive, representative Canadian primary care EMR database which meets or exceeds Canadian privacy requirements and is accessed by state-of-the-art software to build de-identified patient cohorts. D9<br><br>IQVIA Canada EMR (AppleTree) – A Canadian de-identified EMR of 1.2 million individuals from AppleTree Medical Group,[22] of whom approximately 300 000 had at least a single laboratory test. D10<br><br>IQVIA never has access to a patient record or prescription, which identifies the patient. The information collected does not identify any patient; it may include the age and gender of a patient." D12<br><br>A significant opportunity exists to collaborate with other medical institutions and leading organizations to further assemble valuable de-identified databases; unlocking both clinical and commercial potential.<br>Estimated +8M records to structure and monetize. D13<br><br>Secure transfer of de-identified EMR to QI. D18
	Physician data are not de-identified	[The information] may also include the following Personal Information and Professional Services Information relating to the health professional in the context of his or her practice: the name or other identifier, together with the age, gender, office and preferred mailing address, preferred language of communication, hospital affiliations, specialization and year of qualification, of individual health professionals, as well as Prescriber Information. With the consent of the health professional concerned, information concerning diseases diagnosed and treated by the health professional may also be disclosed. D12<br><br>2.1.7 Practice Information means information collected by IQVIA concerning the diagnosis or treatment of diseases by identifiable health professionals. D12

		2.1.9 Professional Services Information means information relating to a health services provider that is about the provision of health services by the provider and identifies the health services provider but does not identify the patient, and includes Practice Information and Prescriber Information as defined in Clauses 2.1.7 and 2.1.8. D12<br><br>3.2.2 This information is processed and analyzed to provide information products in the form of Practice Information, Basic Information or Prescriber Information (in accordance with Clauses 3.3.1.1, 3.3.1.2 and 3.3.1.3 respectively), information in aggregate form, as well as non-identifiable information products. IQVIA offers these products exclusively to healthcare stakeholders in Canada. D12<br><br>3.2.3.1 Where IQVIA collects Practice Information, IQVIA specifies, in a written agreement with the health professional, the purposes for which such information is being collected. D12<br><br>Protected Information covers two general categories of information – Personal Information and Professional Services Information. Personal Information includes Basic Information of a health services professional. Professional Services Information includes Practice Information and Prescriber Information. All of these terms are more specifically defined in Section 2.1 of this code. D12
	Physicians grant consent to provide their identified data and the de-identified patient data to the data broker	Permission granted for secondary use of data by 152 physicians with support from 345 other Health Care Professionals (HCP). D1<br><br>**True or False**: Patients need to provide permission to use EMR data for research<br>If the personal health information has been properly de-identified and the risk of re-identification tested then this is **FALSE**. Physician permission is required. D3<br><br>With the consent of the health professional concerned, information concerning diseases diagnosed and treated by the health professional may also be disclosed. D12
	The data contained detailed information on individual patients (patient-level data)	Primary care EMR database with 2.2 million patient records from Ontario, Canada. D1<br><br>RWD is PATIENT-level data: consumer data, wearables, clinical outcome assessment, lab/biomarkers data, social media data, pharmacy data, hospital data, claims data, prospective and enriched studies, electronic medical and health records. D2<br><br>PATIENT-level data Clinical Outcome Assessments Wearables Consumer data Registries Pharmacy data Electronic medical and health records Prospective and enriched studies Copyright @2018 IQVIA, All Rights Reserved – Real World Evidence – An Industry Perspective Copyright @2018 IQVIA, All Rights Reserved – Real World Evidence – An Industry Perspective Claims data 3 Hospital data Lab/biomarkers data Mortality data Social media data D2<br><br>Drawn from thousands of physicians, it provides the unparalleled geographic coverage necessary for comparing treatment patterns across the country. Patient demographics, clinical variables such as diagnoses and laboratory test results, therapeutic outcomes and treatments are tracked longitudinally. D4<br><br>Key information collected Basic: • Age • Gender • Height • Weight • BMI • Smoking • PFTs, BP, Pulse, Temp • Comorbidities

		Drug Therapy: • Prescribed drug (DIN, ATC-WHO GM) – Molecule, brand & generic name • Manufacturer • Administration • Form • Strength • sic (posology) • Dose • Stop records • First Rx and refills<br>Non-Drug Therapy: • Remedies and aids<br>Diagnosis/Procedures/Lab: • Diagnoses (ICD-9) • Procedures not routinely recorded • Lab test results electronically integrated<br>Other: • Signs & symptoms • Risk factors • Cost & date of treatment • Referrals • Sickness certificate • Telemedicine/ call length<br>D4<br><br>Extra Fields from physician: Ethnicity/SES [socio-economic status], Visuals: Scans/X-rays/ MRI/Ultrasound, Patient Portal Outreach QOL [quality of life] Surveys. D4<br><br>IMS Brogan was able to gather information from Patient Reported Outcome (PRO) surveys as well, which gives quantitative insights on which medications and products patients with diabetes and other disorders are using, their overall satisfaction with the treatments, and self reported quality of life and outcomes. These findings are combined to demonstrate gaps in therapy and need for treatment innovations, as well as demonstrate the value of medications. D6<br><br>IQVIA Canada EMR (AppleTree) – A Canadian de-identified EMR of 1.2 million individuals from AppleTree Medical Group,[22] of whom approximately 300 000 had at least a single laboratory test. D10<br><br>[…] diagnoses, vital signs, lab test results, time off work, drug identification numbers, programs (smoking cessation programs, medication reviews, telemedicine), smoking status, specialist referrals and more. [D15]
	The databases contain a large number of individual patients	Primary care EMR database with 2.2 million patient records from Ontario, Canada. D1<br><br>Statistically robust and easy to access. D3<br><br>Drawn from thousands of physicians, it provides the unparalleled geographic coverage necessary for comparing treatment patterns across the country. Patient demographics, clinical variables such as diagnoses and laboratory test results, therapeutic outcomes and treatments are tracked longitudinally. D4<br><br>The database contains 1.1 million unique patients' anonymized electronic medical records with 7.7 million unique visits. Some records go back as far as the mid-1990s. D4<br><br>The Challenge: Gain access to and securely de-identify up to 5 million Electronic Medical Records (EMRs) to produce quality data for analysis in public health and disease outcome reports or to be shared with manufacturers and researchers. D6<br><br>As a direct result, IMS Brogan now has the potential to access the records of up to 5 million patients from a network of 5,850 providers working in more than 2,600 primary care sites in the Canadian province of Ontario. D6<br><br>Putting the 'T' in RWD T-shaped Evidence Networks reflect the need to consider both breadth and depth when accessing the most appropriate fact base. • RWD sources spanning broad, near population-level cohorts but with limited clinical information about each patient • Clinically rich, deep data for a discrete population of patients, typically with

		coverage of 5-10% of a total market population Ask us about our innovative approaches to enabling clients to build out their own T-shaped networks. D7<br><br>Click here to learn more about IMS Evidence 360 Cohort Builder and access to many data dimensions for ~865,000 patients in a single linked database. D8<br><br>IQVIA Canada EMR (AppleTree) – A Canadian de-identified EMR of 1.2 million individuals from AppleTree Medical Group,[22] of whom approximately 300 000 had at least a single laboratory test. D10<br><br>Founded in 1992, Appletree Medical Group is one of Canada's largest and most diverse multi-specialty medical groups. D11<br><br>MCI OneHealth is one of the largest primary care clinic groups in Canada (25 tech enabled clinics) & a provider of telehealth/virtual care (over 200,000 telehealth visits over the last 8 months). D13<br><br>MCI OneHealth is assembling from its various clinical databases what will eventually be one of the largest de-identified primary care databases in Canada to empower physicians and unlock both clinical and commercial potential. D13<br><br>A quick plug for IMS! • Launched in 2013 using data from 750,000 Canadian EMRs • Integrated lab results • Validated for 6 diseases so far • Over 17 studies delivered • Team includes 2 epis, 4 PhD stats, data scientists, analysts and consultants • Using PARAT de-id software • Partnerships with many academic institutions<br>D 18<br><br>Source: IMS Evidence 360: A database of 850,000 Canadian De-identified longitudinal electronic medical records. Period: June 2013 to May 2014. D20
	The data are longitudinal and show the whole patient pathway overtime	The database contains 1.1 million unique patients' anonymized electronic medical records with 7.7 million unique visits. Some records go back as far as the mid-1990s. D4<br><br>Furthermore, the power of longitudinal EMR patient data is that it permits a greater understanding of the relationship between testing, diagnosis, treatment and outcomes, in the investigation of many disease states beyond gonorrhea. D17<br><br>Encryption methodologies allow for de-identification, blending and linking data across various datasets, illustrating the full patient journey. D15<br><br>Source: IMS Evidence 360: A database of 850,000 Canadian De-identified longitudinal electronic medical records. Period: June 2013 to May 2014. D20<br><br>For example, real-world evidence studies demonstrate practical and clinical efficacies, which we believe require the aggregation and integration of large clinical data sets across all care settings, types of therapies and patient cohorts. Longitudinal studies require analysis of non-identified patient diagnoses, treatments, procedures and laboratory test results to identify types of patients that will likely best respond to particular therapies. D24

	In some cases, the data can be linked at patient-level across multiple datasets	Diagram describing data sources, linked at patient-level. D2<br><br>To bring deeper insights into patient treatment and outcomes, and better understand the full patient pathway, QuintilesIMS has developed methodologies to enrich existing EMR datasets by linking them to other datasets or prospectively collected information. D4<br><br>Putting the 'T' in RWD T-shaped Evidence Networks reflect the need to consider both breadth and depth when accessing the most appropriate fact base. • RWD sources spanning broad, near population-level cohorts but with limited clinical information about each patient • Clinically rich, deep data for a discrete population of patients, typically with coverage of 5-10% of a total market population Ask us about our innovative approaches to enabling clients to build out their own T-shaped networks. D7<br><br>Data linkage • Understanding the full patient pathway through integrating datasets at the record level (eg, IMS RWD integrated offerings in the USA, Pygargus methodology in Scandinavia). D7<br><br>IMS Brogan enables this process by bringing together multiple data sources, an intelligent technology layer between the raw data and the ultimate analytical need, and innovative researchers and methodologies. D9<br><br>Encryption methodologies allow for de-identification, blending and linking data across various datasets, illustrating the full patient journey. D15<br><br>A quick plug for IMS! • Launched in 2013 using data from 750,000 Canadian EMRs • Integrated lab results • Validated for 6 diseases so far • Over 17 studies delivered • Team includes 2 epis, 4 PhD stats, data scientists, analysts and consultants • Using PARAT de-id software • Partnerships with many academic institutions<br>D 18
	The data are available in a variety of formats	Delivered in a variety of formats: Data query tool and a one-year data license to the full database enabling data extraction for import into SAR / R / SPSS / Python, etc. D4<br><br>With Privacy Analytics' software, IMS Brogan is able to de-identify patient data to meet the needs of specific studies. Clients can then gain direct access to run their own analysis of the data to better understand the patient population that their products are treating, evaluate unmet needs, inform recruitment for clinical trials or evaluate comparative treatments that are available. D6<br><br>Data Access: How can the data be accessed?<br>IMS Evidence 360 Cohort Builder enables the researcher to simply build cohorts using inclusion exclusion criteria on multiple parameters. D9
	These primary care databases have a lot more information than the databases the pharmaceutical industry had access to before	Not just the domain of the academic researcher anymore, data is available to the pharmaceutical industry. D3<br><br>Unique in Canada, this database represents the only commercially available EMR research database for the pharmaceutical industry. The database contains 1.1 million unique patients' anonymized electronic medical records with 7.7 million unique visits. Some records go back as far as the mid-1990s. Update frequency is available monthly or quarterly. D4

		Before working with Privacy Analytics, IMS Brogan had access to prescription and claims data, which had much less patient identifying information in it, but as a result, lacked the rich analytic value of EMR data. When EMR data is responsibly de-identified, it can be used to create customized datasets that enable highly detailed performance analytics reporting and research. D6<br><br>This data is now available from IMS Brogan for the Canadian market and studies can be undertaken with the Canadian RWE [Real World Evidence] team as part of the global RWE [Real World Evidence] Centre of Excellence. D15
	The data are valuable because it generates clinical evidence – "real world evidence".	Real-World Evidence (RWE): Insights generated from RWD using appropriate scientific and/or generated commercial analytics with the intention to support a claim or belief to produce evidence for multiple stakeholders. D3<br><br>EMR [electronic medical record] data has been used by NICE and other HTA bodies in Europe for a very long time and is considered the gold standard for Real World Evidence research. D3<br><br>Real-World EMR-Canada information is anonymized patient records collected from Patient Management software used by GPs and select specialists within their community-based specialist office during an office visit to document patients' clinical records. D4<br><br>As scientifically credible, anonymized patient-level data becomes more accessible around the globe, real-world evidence (RWE) is becoming the new currency in healthcare. Healthcare decisions can now be better informed in the real world, enabling physicians and payers to make decisions based on how the drug works with the patient in the doctor's office and what impact the medication is having on the care of the patient. D4<br><br>Real-World EMR-Canada is the most comprehensive, clinically rich source of electronic medical records available in the country. D4<br><br>As scientifically credible, anonymized patient-level data becomes more accessible around the globe, real-world evidence (RWE) is becoming the new currency in healthcare. D8<br><br>Observational data derived from clinical practice is becoming increasingly important to answer questions that cannot be addressed in Randomized Clinical Trials. D9<br><br>Patient outcomes are becoming the most meaningful currency for healthcare decision making. The role of real-world evidence (RWE) in assessing these endpoints has elevated its importance on every major life sciences company agenda, putting health economics & outcomes research (HEOR), epidemiology and drug safety at the heart of the desired transformation. D9<br><br>The objectives of this study were to understand a gout population in terms of demographics, clinical characteristics, healthcare utilization and costs versus a gout-free population. D14<br><br>[T]here has been recognition that RWE [real world evidence] has broader applications than research, and the term RWE is often interchanged with Real World Data (RWD). These broader applications go beyond the notion of public

		evidence, but apply the same techniques and data to enable internal business decision-making in pharma and by payers. D15<br><br>Real World Evidence can be used to help support arguments from RCTs throughout the market access process. D15<br><br>RWE Can Improve Competitive Position Throughout a Brand's Life Cycle The complex logistics involved in fulfilling RWE requirements call for a heterogeneous approach. Fundamentally, early planning is required to ensure the quality of responses, rational RWE spend throughout the life cycle, and proactive efforts to differentiate and position a brand. D15<br><br>What questions might be important to ask when we have decided that using real world evidence is the best option? D18<br><br>"RWE is the New Black: A Perspective from Industry" − Julia Brown, Vice President, Government Affairs and Market Access, Janssen Inc. D20<br><br>Routinely collected data within health systems is increasingly being viewed as a means to increase payer certainty by conducting assessments of performance in the "real world." D20<br><br>The last 10 years in Canada has seen a shift toward the use of real world evidence in some areas. As this use of real-world evidence becomes routine, it will have a real impact on payers and the HTA [Health Technology Assessment] bodies that support them, as well as providers, patients and industry. D20<br><br>Trust the evidence. It is easy to criticize the data. Big data will drive the future of our health system. • Are our policies progressive enough to match those in Europe? Or are we going to be left behind in Canada? D20<br><br>In order to derive valuable insights from existing and expanding sources of information, clients need access to statistically significant data sets organized into databases that can be queried and analyzed. For example, real-world evidence studies demonstrate practical and clinical efficacies, which we believe require the aggregation and integration of large clinical data sets across all care settings, types of therapies and patient cohorts. D25
	Electronic medical records data are a valuable form of real-world data	EMR [electronic medical record] data has been used by NICE and other HTA bodies in Europe for a very long time and is considered the gold standard for Real World Evidence research. D3<br><br>Real-World EMR-Canada information is anonymized patient records collected from Patient Management software used by GPs and select specialists within their community-based specialist office during an office visit to document patients' clinical records. D4<br><br>Real-World EMR-Canada is the most comprehensive, clinically rich source of electronic medical records available in the country. D4
	The evidence is as valuable as evidence generated from clinical trials	The vast majority of data around patient exposure to interventions occurs outside of traditional clinical trials. D2<br><br>RWE is relevant and critical, since the real world does not consist of "ideal patients." D2<br><br>Real World Data (RWD): patient-level data not collected in randomized controlled trials D2.

		As scientifically credible, anonymized patient-level data becomes more accessible around the globe, real-world evidence (RWE) is becoming the new currency in healthcare. Healthcare decisions can now be better informed in the real world, enabling physicians and payers to make decisions based on how the drug works with the patient in the doctor's office and what impact the medication is having on the care of the patient. D4<br><br>As scientifically credible, anonymized patient-level data becomes more accessible around the globe, real-world evidence (RWE) is becoming the new currency in healthcare. D8<br><br>Observational data derived from clinical practice is becoming increasingly important to answer questions that cannot be addressed in Randomized Clinical Trials. D9<br><br>The goal is not to replace RCTs [with RWE], which are considered the gold standard of evidence, but to complement them. D15<br><br>The secondary factors highlighted now as additional points for reviewing the pricing of products are: ○ Route of administration ○ Patient convenience }Compliance improvements leading to improved therapeutic efficacy ○ Caregiver convenience }Time required to achieve the optimal therapeutic effect ○ Duration of usual treatment course ○ Success rate }Percentage of affected population treated effectively ○ Disability avoidance/savings. Many of these factors are not available from clinical trials and will rely on RWE data both in Canada and in other countries to deliver the required evidence. D15<br><br>What questions might be important to ask when we have decided that using real world evidence is the best option? D18<br><br>"RWE is the New Black: A Perspective from Industry" − Julia Brown, Vice President, Government Affairs and Market Access, Janssen Inc. D20
	The international pharmaceutical industry values health data and data technologies	Build upon our extensive client relationships. We have a diversified base of over 5,000 clients in over 100 countries, and through the Merger have expanded our client value proposition to address a broader market for research and development and commercial operations which we estimate to be $230 billion in 2016. Through the combined offerings of research and development and commercial services we built a platform that allows us to be a more complete partner to our clients. D21<br><br>Despite expected accelerating growth in the global life sciences market, we believe our clients will face increased operating margin pressure due to their changing product mix, pricing and reimbursement challenges, and rising costs of compliance. Product portfolios for life sciences companies have shifted toward specialty products with lower peak market sales potential than traditional primary care medicines. D22<br><br>IQVIA creates intelligent connections across all aspects of healthcare through its analytics, transformative technology, big data resources and extensive domain expertise. IQVIA Connected Intelligence™ delivers powerful insights with speed and agility — enabling customers to accelerate the clinical development and commercialization of innovative medical treatments that improve healthcare outcomes for patients. With approximately 70,000 employees, we conduct operations in more than 100 countries. D25

		Real-World Evidence and connected health: Total addressable market of approximately $80 billion based on 2020 sales that consists of two relatively equal parts. First, the market for Real-World Evidence of approximately $40 billion includes traditionally defined analytic platforms and implementation, medical and scientific analytic services, observation studies and market access. Second, the market for connected healthcare of approximately $40 billion includes areas such as revenue cycle management, payer analytics and clinical decision support services. D25
	Gain regulatory approval	Global Market Research • Market opportunity assessments • Event-based forecasts • Launch tracking, KPIs Linkage of IMS RWD Diabetes with client data Integrated client RCT with multiple IMS RWD assets to support pragmatic trials Analysis of IMS RWD Claims–US to support product value Analyzed IMS RWD Claims–US to show value of a branded product versus generics of a competing molecule for specific patient profile HEOR Regulatory Affairs Custom studies in support of regulatory filings, eg, • Burden of illness • Value dossiers • Compliance & persistence studies • Pharmacoepidemiology & drug safety D7<br><br>Typical examples of types of reports that can be undertaken from the IMS Evidence 360 Cohort Builder<br>FIGURE 5 RWE OFFERING KEY COMPONENTS<br>Burden of Illness<br>• Prepare a Test and Control Cohort of patients • Evaluate the direct, indirect, and societal costs to treat each cohort<br>• MD/ER visits, hospitalizations, diagnostic tests, patient monitoring, productivity<br>APPLICATIONS<br>• Define the unmet need of a disease • Quantify the impact of a disease on Canadians • Raise awareness of the importance of improving disease management through publication<br>• Facilitate discussion with payer and policy makers<br>Cost Effectiveness<br>• Lab results pre and post treatment • Outcomes based on labs, diagnosis and Tx • Drug cost • Other health care costs • Persistence & compliance • Dose escalation • Incidence & prevalence • Lines of therapy<br>• Supplement evidence package for CDR [Canadian Agency for Drugs and Technologies in Health (CADTH) Common Drug Review] and PCPA [pan-Canadian Pharmaceutical Alliance]<br>• Understand the economic value of an individual treatment<br>D9<br><br>Data Application: What can it be used for?<br>IMS EVIDENCE 360 COHORT BUILDER ENABLES FAST INSIGHTS TO DATA NOT READILY AVAILABLE PREVIOUSLY IN CANADA<br>Example: Understanding HbA1c levels of patients taking diabetes medications and deeper insights into patients taking DPP4 medication - Patient Diagnostics from IMS Evidence 360 Cohort Builder, January 2013 – December 2013.<br>D9

		In conclusion, we describe the employment of a machine learning model to predict the progression to diabetes in over 1 million persons with pre-diabetes during an average 5 years of follow up. The model may be incorporated in the EMRs [electronic medical records] and alert for screening intervals or enable selection of high-risk individuals for interventional programmes, demonstrating a better PPV [positive predictive value] than current alternatives. D10<br><br>RWD EMR-Canada has formed the basis of many studies and peer-reviewed scientific publications. The Canadian team undertook 156 studies in 2016 with focus on Market Access, HEOR, Patient Support Program design and analysis, coverage with evidence development and Real World Data Insights for HTA. D15<br><br>Real World Evidence can be used to help support arguments from RCTs throughout the market access process. D15<br><br>The last 10 years in Canada has seen a shift toward the use of real world evidence in some areas. As this use of real-world evidence becomes routine, it will have a real impact on payers and the HTA [Health Technology Assessment] bodies that support them, as well as providers, patients and industry. D20
	Demonstrate cost effectiveness to regulators and payers	Value dossiers D7<br><br>Raise awareness of the importance of improving disease management through publication<br>• Facilitate discussion with payer and policy makers<br>Cost Effectiveness<br>• Lab results pre and post treatment • Outcomes based on labs, diagnosis and Tx • Drug cost • Other health care costs • Persistence & compliance • Dose escalation • Incidence & prevalence • Lines of therapy<br>• Supplement evidence package for CDR [Canadian Agency for Drugs and Technologies in Health (CADTH) Common Drug Review] and PCPA [pan-Canadian Pharmaceutical Alliance]<br>• Understand the economic value of an individual treatment<br>D9<br><br>Routinely collected data within health systems is increasingly being viewed as a means to increase payer certainty by conducting assessments of performance in the “real world”. D20<br><br>The last 10 years in Canada has seen a shift toward the use of real world evidence in some areas. As this use of real-world evidence becomes routine, it will have a real impact on payers and the HTA [Health Technology Assessment] bodies that support them, as well as providers, patients and industry. D20
	Determine pricing	The secondary factors highlighted now as additional points for reviewing the pricing of products are: ○ Route of administration ○ Patient convenience }Compliance improvements leading to improved therapeutic efficacy ○ Caregiver convenience ○ Time required to achieve the optimal therapeutic effect ○ Duration of usual treatment course ○ Success rate }Percentage of affected population treated effectively ○ Disability avoidance/savings.<br>Many of these factors are not available from clinical trials and will rely on RWE data both in Canada and in other countries to deliver the required evidence. D15
	Understand physician behaviour	The evidence [from the EMR data] is used for [market] access purposes and for better understanding the decision points by physicians D3

		Key applications<br>[EMR data] has a variety of research applications, including: • Pharmacoepidemiology • Drug safety & risk management • Pharmacovigilance • Health economics & outcomes research • Compliance & persistence • Pharmaceutical guidelines • Prescribing behaviour • Drug usage D4<br><br>Pharmaceutical companies use the information to educate prescribers and to better understand their information needs with respect to effective and cost efficient prescribing practices and new products and therapies. D12
	Market products	Global Market Research • Market opportunity assessments • Event-based forecasts • Launch tracking, KPIs Linkage of IMS RWD Diabetes with client data Integrated client RCT with multiple IMS RWD assets to support pragmatic trials Analysis of IMS RWD Claims–US to support product value Analyzed IMS RWD Claims–US to show value of a branded product versus generics of a competing molecule for specific patient profile HEOR Regulatory Affairs Custom studies in support of regulatory filings, eg, • Burden of illness • Value dossiers • Compliance & persistence studies • Pharmacoepidemiology & drug safety D7<br><br>• Often published in journals, at conferences to build credibility and raise awareness. D9<br><br>Raise awareness of the importance of improving disease management through publication<br>• Facilitate discussion with payer and policy makers<br>Cost Effectiveness<br>• Lab results pre and post treatment • Outcomes based on labs, diagnosis and Tx • Drug cost • Other health care costs • Persistence & compliance • Dose escalation • Incidence & prevalence • Lines of therapy<br>• Supplement evidence package for CDR [Canadian Agency for Drugs and Technologies in Health (CADTH) Common Drug Review] and PCPA [pan-Canadian Pharmaceutical Alliance]<br>• Understand the economic value of an individual treatment<br>D9<br><br>Pharmaceutical companies use the information to educate prescribers and to better understand their information needs with respect to effective and cost efficient prescribing practices and new products and therapies; to obtain participation in clinical trials of new products; to facilitate drug warnings and recalls; and to market their products. D12<br><br>Real World Evidence for the Canadian Pharmaceutical Market. D15<br><br>This data is now available from IMS Brogan for the Canadian market and studies can be undertaken with the Canadian RWE [Real World Evidence] team as part of the global RWE [Real World Evidence] Centre of Excellence. D15<br><br>RWE Can Improve Competitive Position Throughout a Brand's Life Cycle The complex logistics involved in fulfilling RWE requirements call for a heterogeneous approach. Fundamentally, early planning is required to ensure the quality of responses, rational RWE spend throughout the life cycle, and proactive efforts to differentiate and position a brand. D15<br><br>The complex logistics involved in fulfilling RWE requirements call for a heterogeneous approach. Fundamentally, early planning is required to ensure the quality of responses, rational RWE spend throughout the life cycle, and proactive efforts to differentiate and position a brand. Success will depend on: Careful and routinely updated analysis of current

		and future comparators, drilling down to actual/perceived differentiation on specific claims, to enable tactical planning of individual studies to address those differentiation claims<br>A long-term view of supporting a product's claims with analyses of specific data sources, based on early inventory/vetting of databases that could support RWE studies and specific plans to address gaps by creating data sources where they are unavailable<br>A tactical evidence-generation plan that enables prioritization of data acquisition and analyses – both to anticipate external demands and to maximize the execution of a differentiation strategy. D15
	Data are valued in other jurisdictions/countries as well	Build upon our extensive client relationships and leverage our global presence. We have a diversified base of over 10,000 clients in over 100 countries and have expanded our client value proposition to address a broader market for research and development and commercial operations which we estimate to be more than $260 billion in 2020. Through the combined offerings of research and development and commercial services we built a platform that allows us to be a more complete partner to our clients. D23<br><br>IQVIA creates intelligent connections across all aspects of healthcare through its analytics, transformative technology, big data resources and extensive domain expertise. IQVIA Connected Intelligence™ delivers powerful insights with speed and agility — enabling customers to accelerate the clinical development and commercialization of innovative medical treatments that improve healthcare outcomes for patients. With approximately 70,000 employees, we conduct operations in more than 100 countries. D25
**Claiming social legitimacy**	The databases provide societal benefit.	RWE is here to stay, and holds the promise to greatly improve healthcare decision-making. D2<br><br>Can we reduce the $2.1B development cost for a drug today, and ultimately lead to lower drug prices? What can we do to address $500 Billion in avoidable annual medical costs globally because medicines are not used responsibly? Can we find the 20-40% of Canadians diabetics who are undiagnosed and untreated? Can we increase adherence of the 60% of patients on chronic therapy who are not refilling their prescription after 6 months? Can we prevent the 4,000-8,000 Canadian deaths due to diagnostic errors every year? D2<br><br>Over the years, IQVIA (Canada) has worked with countless health professionals, academic institutions, pharmaceutical manufacturers and governments to provide evidence-based information to support advances in healthcare. The unique value of that information has been unlocked by these stakeholders - to increase public awareness, help shape public policy and improve the well-being of millions of Canadians. [D5]<br><br>Access to quality datasets for academic research institutes and medical manufacturers provides better understanding of the patient population, including the ability to evaluate unmet needs, compare cost and effectiveness of existing treatments, find connections between diseases and improve healthcare overall. D6<br><br>Healthcare decisions can now be better informed in the real world, enabling physicians and payers to make decisions based on how the drug works with the patient in the doctor's office and what impact the medication is having on the care of the patient. D8

		We conduct anonymised real-world evidence studies that help identify best practices and lead to better health outcomes in our communities. We are particularly proud of the measurable, direct and positive health impact this research has had on the communities we serve. D11<br><br>Empowering patients and doctors with advanced technologies to increase access, improve quality,and reduce the costs of healthcare. D13<br><br>MCI OneHealth is assembling from its various clinical databases what will eventually be one of the largest de-identified primary care databases in Canada to empower physicians and unlock both clinical and commercial potential. [D13]
	The creation and use of the databases present minimal risk to privacy	None of the patients analyzed had their identity or medical records disclosed for the purposes of this study, and only anonymized patient-level data were accessed. Because no identifiable protected health information was extracted or accessed during the course of this study, no institutional review board review or approval was required. D2<br><br>Diagram describing the privacy “layers.” D4<br><br>The Solution: Build a customized process using Privacy Analytics’ software to de-identify EMR data so that it can be safely utilized without putting patient privacy at risk. D6<br><br>In order to secure a partnership with an EMR vendor and gain access to EMR data, IMS Brogan had to give assurance that patient privacy would not be at risk. A commitment to use Privacy Analytics’ de-identification expertise and software - the only de-identification software that applies the Expert Determination methodology - provided the assurance that the EMR vendor needed. D6<br><br>Privacy Analytics’ software provided IMS Brogan with the only way to achieve a scalable, HIPAA compliant approach to accessing and sharing EMR data for important secondary purposes. D6<br><br>All stakeholders need to provide input on what is most important to them, be it data utility or privacy. It’s not easy to balance the needs of everyone involved, but good communication and a commitment to producing useful data that keeps the risk of re-identification low is all you really need to get started. It’s not an easy negotiation— and it may be iterative—but it is an important negotiation to have. D6<br><br>And we ensure that quality management and patient privacy are never compromised. D7<br><br>IMS Health provides groundbreaking, built-for-purpose technology applications to enable healthcare RWE, and we ensure that quality management and patient privacy are never compromised. D8<br><br>IMS Brogan has addressed this need with a comprehensive, representative Canadian primary care EMR database which meets or exceeds Canadian privacy requirements and is accessed by state-of-the-art software to build de-identified patient cohorts. D9

		As a global leader in protecting individual patient privacy, QuintilesIMS uses healthcare data to deliver critical, real-world disease and treatment insights. Through a wide variety of privacy-enhancing technologies and safeguards, QuintilesIMS protects individual privacy while managing information to drive healthcare forward. D9<br><br>The information collected does not identify any patient; it may include the age and gender of a patient. It may also include Protected Information about the health professional in the context of his or her practice: the name or other identifier, age, gender, office and mailing address, hospital affiliations, specialization and year of qualification, and information concerning diseases diagnosed and treated by them and drugs dispensed under their prescriptions. D11<br><br>Practice Information means information collected by IQVIA concerning the diagnosis or treatment of diseases by identifiable health professionals. D11<br><br>Professional Services Information means information relating to a health services provider that is about the provision of health services by the provider and identifies the health services provider but does not identify the patient, and includes Practice Information and Prescriber Information as defined in Clauses 2.1.7 and 2.1.8. D11<br><br>Where IQVIA collects Practice Information, IQVIA specifies, in a written agreement with the health professional, the purposes for which such information is being collected. D11<br><br>Where IQVIA collects Practice Information, IQVIA's staff involved in the collection is trained to explain to the health professional, at the time of the written agreement referred to in Clause 3.2.3.1, the purposes for which the information is being collected. D11<br><br>IQVIA never has access to a patient record or prescription, which identifies the patient. The information collected does not identify any patient; it may include the age and gender of a patient. [D12]<br><br>The steps that need to be undertaken to take EMR data and build an RWE data warehouse are complex and are governed by strict privacy guidelines and regulations. Once this process has been defined and the data warehouse built, the "patient dimensions" can be extracted and used to undertake Real World Evidence studies. The many inputs to the process are shown in Figure 2. D15<br><br>There is ongoing concern from privacy advocates, regulators and others regarding data protection and privacy issues, and the number of jurisdictions with Privacy Laws has been increasing. Also, there are ongoing public policy discussions regarding whether the standards for de-identified, anonymous or pseudonymized health information are sufficient, and the risk of re-identification sufficiently small, to adequately protect patient privacy. These discussions may lead to further restrictions on the use of such information. There can be no assurance that these initiatives or future initiatives will not adversely affect our ability to access and use data or to develop or market current or future services. D22<br><br>Our use of a wide range of privacy and security safeguards protect non-identified patient-level medical claims, prescriptions, electronic medical records, genomics, patient reported outcome and social media data. D25<br><br>Laws and expectations relating to privacy continue to evolve, and we continue to adapt to changing needs. For example, the definition of "personally identifiable information" and "personal data" continues to evolve and broaden and many

		new laws and regulations are being enacted. In addition, certain established programs have been (or are at risk of being) declared invalid (such as the EU-U.S. Privacy Shield framework that operated for several years but was struck down by the European Court of Justice in July, 2020), so that this area remains in a state of flux. Changes to these programs may adversely impact our ability to provide services to our clients or develop new products or services. Federal, state and foreign governments are contemplating or have proposed or adopted new Privacy Laws or modifications to existing Privacy Laws, including by amendment, replacement or interpretation through judicial or administrative decisions. New or modified Privacy Laws might, among other things, require us to implement new security measures and processes or bring within the scope of the Privacy Law other data not currently regulated, each of which may require substantial expenditures or limit our ability to offer some of our services. Additionally, changes in Privacy Laws may limit our data access, use and disclosure, and may require increased expenditures by us or may dictate that we not offer certain types of services. Any of the foregoing may have a material adverse impact on our ability to provide services to our clients or maintain our profitability. D25
